# The Influence of Heat Stress on Chicken Immune System and Mitigation of Negative Impacts by Baicalin and Baicalein

**DOI:** 10.3390/ani13162564

**Published:** 2023-08-09

**Authors:** Vladimir Zmrhal, Andrea Svoradova, Eva Venusova, Petr Slama

**Affiliations:** 1 Laboratory of Animal Immunology and Biotechnology, Department of Animal Morphology, Physiology and Genetics, Faculty of AgriSciences, Mendel University in Brno, 613 00 Brno, Czech Republic; vladimir.zmrhal@mendelu.cz (V.Z.); andrea.svoradova@mendelu.cz (A.S.); eva.venusova@mendelu.cz (E.V.); 2 NPPC, Research Institute for Animal Production in Nitra, 951 41 Luzianky, Slovakia

**Keywords:** heat stress, broiler chicken, immune system, baicalin, baicalein

## Abstract

**Simple Summary:**

Research in the field of heat stress in poultry focusing on additives with immunomodulatory properties is reviewed here. Modulations of the immune system components are strongly associated with the negative impacts of heat stress on poultry production. The research reported the anti-inflammatory and antioxidant properties of baicalin and baicalein to support their benefits for poultry under heat-stress conditions. Therefore, these two flavonoids can mitigate the negative impacts of heat stress on the immune system and improve poultry health and performance under stressful conditions.

**Abstract:**

Heat stress (HS) in poultry husbandry is an important stressor and with increasing global temperatures its importance will increase. The negative effects of stress on the quality and quantity of poultry production are described in a range of research studies. However, a lack of attention is devoted to the impacts of HS on individual chicken immune cells and whole lymphoid tissue in birds. Oxidative stress and increased inflammation are accompanying processes of HS, but with deleterious effects on the whole organism. They play a key role in the inflammation and oxidative stress of the chicken immune system. There are a range of strategies that can help mitigate the adverse effects of HS in poultry. Phytochemicals are well studied and some of them report promising results to mitigate oxidative stress and inflammation, a major consequence of HS. Current studies revealed that mitigating these two main impacts of HS will be a key factor in solving the problem of increasing temperatures in poultry production. Improved function of the chicken immune system is another benefit of using phytochemicals in poultry due to the importance of poultry health management in today’s post pandemic world. Based on the current literature, baicalin and baicalein have proven to have strong anti-inflammatory and antioxidative effects in mammalian and avian models. Taken together, this review is dedicated to collecting the literature about the known effects of HS on chicken immune cells and lymphoid tissue. The second part of the review is dedicated to the potential use of baicalin and baicalein in poultry to mitigate the negative impacts of HS on poultry production.

## 1. Introduction

Today, heat stress (HS) is increasing in importance due to global warming, and its impacts on poultry production are well known. HS causes performance parameters to deteriorate in both broiler chickens [1] and laying hens [2]. Animal performance is, of course, a determining factor of profitability in poultry husbandry. In recent years, data have been collected, and mechanisms described as to how HS negatively influences weight gain and meat quality in meat-type chickens and disrupts eggshell quality and egg production in laying hens [3,4]. Additionally, HS complicates health management in poultry even as poultry species constitute a rich source of zoonotic diseases [5]. Stress has immunosuppressive potential as a general matter, and many studies in poultry have proven that HS causes dysregulation of the immune system in chickens [6,7,8]. Most negative impacts of HS are associated with oxidative stress, which means the overproduction of reactive oxygen species (ROS) beyond the antioxidant capacity of the organism [9]. The most devastating impacts of oxidative stress occur on the chicken gut, which is the largest area of the body exposed to the environment and the site of most of the immune cells [10]. Therefore, one of the most important factors affecting the poor performance of the chicken under HS conditions is the stimulation of inflammatory processes in the chicken gut [11]. The importance of poultry health management is increasing in today’s world which is endangered by global warming and the coronavirus (COVID-19) pandemic. Therefore, this review focuses on current knowledge of the impacts of heat and oxidative stress on chicken immune cells and lymphoid tissue. However, very little is known about the impacts of HS on individual immune cell phenotypes; therefore, this review summarizes the established effects of heat stress on these cells in chickens. Phytochemicals constitute a current avenue to mitigating HS in poultry. Currently, intensive research in the field of phytogenic additives preparation occurs; therefore, there is a need to collect information about phytogenic additives with the most promising effects to mitigate HS. Many phytochemicals have anti-inflammatory and antioxidative properties, and some authors have introduced possible ways that these plant-based compounds can decrease inflammation and ROS load in the chicken organism [9,12]. In recent years, the intensive study of baicalin and its aglycon baicalein has produced very promising results in relation to mitigating the negative impacts of stressors on poultry. Baicalin and baicalein successfully decrease the impacts of stress in previous studies. Therefore, the second part of the review is dedicated to describing a possible way to mitigate HS in poultry using baicalin and baicalein.

Information for this review was obtained by searching specific keywords on WOS and PUBMED: heat stress, stress response, heat stress chicken, heat stress poultry, heat stress poultry performance, heat stress food safety, oxidative stress, heat stress immune response, dendritic cells heat stress chicken, macrophages heat stress chicken, B lymphocytes heat stress chicken, T lymphocytes heat stress chicken, heterophil heat stress chicken, baicalin stress chicken, baicalein stress chicken.

## 2. Influence of Heat Stress on Performance Parameters

Stressful conditions cause deterioration of the growth rate of broiler chickens and laying performance. A recent meta-analysis gathered relevant studies to show the influence of HS on the performance parameters of broiler chickens. Results showed a significant decrease in feed intake, body weight gain, and mortality and an increase in feed conversion ratio, due to HS. Nevertheless, most of the studies (80%) included in the meta-analysis studied chronic HS, which has a more aggravating effect then acute HS [13]. Earlier meta-analysis studied the effect of chronic HS on quantitative and qualitative parameters of laying performance. HS was disrupted predominantly by quantitative parameters because there were observed effects on shell strength, daily feed intake, egg mass, and egg production to HS. In the case of Haugh units’ yolk, and albumen proportion, almost no influence of HS was observed. HS works differently depending on age and genotype, which was also shown in the meta-analysis [14]. Energy expenditure due to HS causes organism exhaustion and with increasing age (28 to 42 days) broilers, with increased negative impacts of chronic HS on body weight gain, feed intake, and feed conversion ratio compared to acute HS [15]. Generally, HS worsens the growth of chickens, and the main reason can be attributed to changes in intestine physiology. Intestine morphology seems to be very sensitive to HS because if the chickens were treated only with cyclic HS, smaller intestines and shorter villi were found [16]. Consequently, reduced nutrient absorption area and modulated nutrient transporter gene expression probably account for the impaired performance during HS [17]. Restriction in dietary protein further distinctly reduces the availability of amino acids and this, in turn, reduces the growth of chickens [15]. Therefore, impaired poultry performance by HS is a well-documented phenomenon.

## 3. Systemic Response of Chicken Organism to Heat Stress

Unlike mammals, chickens have limited thermoregulation due to their absence of sweat glands. Nevertheless, birds under high ambient temperatures have developed a system of thermoregulatory behavior comprising elevated wings, panting, and vasodilatation of blood capillaries located in the body periphery [18]. Under stressful conditions, activation of the hypothalamic–pituitary axis occurs. The hypothalamus releases a corticotrophin-releasing hormone, thereby causing the production of adrenocorticotropic hormone in the pituitary gland, which subsequently stimulates the release of corticosterone from the adrenal cortex. In the adrenal medulla, HS stimulates the production of epinephrine and norepinephrine [19]. Administration of corticosterone at several concentrations gradually decreases body weight and spleen weight. Additionally, corticosterone has been shown to reduce antibody titers against sheep red blood cells and their pro-inflammatory properties were presented as increasing levels of blood heterophils, which is a typical symptom of stress [20]. Corticosterone ensures the gain of energy for the “flight or fight” reaction in the chicken body by stimulating glycolysis and glycogenolysis, which increases energy expenditure. Gluconeogenic amino acids are a source for glycolysis, and chickens under chronic HS mobilize these amino acids from the muscle tissue. In doing so, corticosterone retards muscle development and growth performance in chickens [21]. Simultaneously, meat quality also is affected negatively by HS. Glycogenolysis causes glucose to be converted into large quantities of lactic acid, which decrease the pH of meat and, in combination with higher body temperature, causes denaturation of sarcoplasmic proteins and then a decrease in water-holding capacity. In muscles, meanwhile, metabolic acidosis also occurs in heat-stressed chickens due to increased panting and CO_2_ exhalation [22]. In a systemic response to HS, the upregulation of heat shock proteins (HSP) is the first response to increased temperatures. The HSP family is a group of proteins responsible for protecting proteins against denaturation caused by high temperatures. Heat shock transcription factors (HSTF) play a crucial role in inducing the expression of HSP genes. While under thermoneutral conditions HSTF is linked with HSP in the cytoplasm, heat stimulus causes HSP to be released from the HSTF and translocate to the nucleus and activate the expression of HSP-related genes [23]. Within the HSTF family, the most important seems to be HSTF 1 and HSTF 3. HSTF 1 is activated under low temperatures, but HSTF 3 activation occurs with increased environmental temperature [24]. The HSP family is divided based on molecular weight. HSP70 is among the most conserved and extensively studied [25]. In chicken immune cells, increased HSP 70 expression has been observed at high ambient temperatures of about 45 °C [26]. Activation of HSP is also connected with pro-inflammatory conditions under HS, because nuclear factor-κβ (NF-κβ) has been shown to induce cellular defense mechanisms by expression of HSP [27]. HSP is highly expressed mainly in the initial phase of HS. Siddiqui et al. [28] found HSP 70 and HSP 60 to be increased after heat exposure in chicken intestines but then decrease linearly during 12 h after heat exposure. Expression of HSP is highly tissue-dependent, and it is driven by the importance of tissue in maintaining homeostasis. Therefore, the highest expression of HSP 70 within HS broiler chickens has been observed in the brain compared to in the liver and muscles [29]. Production of HSP differs in various tissue types. Xie et al. [30] observed a more pronounced expression of HSP and HSTF in the heart and liver compared to in the muscles of heat-stressed laying hens. Subsequently, HS stimulates strong upregulation of antioxidant enzymes, such as superoxide dismutase, glutathione peroxidase, and catalase, under conditions of HS that are followed by upregulation of such immune-related genes as for the pro-inflammatory cytokines IL-1β, IL-6, and TNF-α, as well as toll-like receptors (TLRs) [8]. The effects of HS on both oxidative stress and the immune system will be discussed below. Examining the influence of HS on nutrient digestion and intestinal microbiota are relatively novel approaches. Nutrient transporter genes for various nutrients are differently expressed during HS. Sun et al. [31] found that amino acid transporters LAT1, CAT1, r-BAT, and PePT-1 were not significantly affected by HS. On the other hand, Habashy et al. [32] report that the amino acids transporters SNAT1, SNAT2, SNAT7, TAT1, and b0 were downregulated in the ileum and musculus pectoralis major. Fatty acids binding proteins consist of a group of fatty acid transporters, and their expression is downregulated in the chicken intestine during HS [33]. As a main source of energy, glucose plays the most important role in metabolism because energy expenditure in chickens increases under HS [34]. Glucose transporter (GLUT) and sodium-dependent glucose transporter (SGLT1) are responsible for glucose absorption in the intestine, and HS has a significant effect on their expression. In the initial phase of HS, SGLT1 is downregulated in the intestine [33]. However, its expression has been observed to remain unchanged in the musculus pectoralis major and intestine after 12 days of exposure to HS [17]. These variations could be attributed to adaptation to HS or decreased feed intake.

The microbiota–gut–brain axis is a novel term in connection with factors produced by the brain and which can affect the intestinal microbiome and gut function. Likewise, the gut and microbiome can influence the brain and mental conditions. In poultry, there exists some evidence of hypothalamic–pituitary axis activation in relation to microbiome and gut functions. HS influences the composition of the host microbiome. Members of the phylum Firmicutes are Gram-positive bacteria including the genera Bacillus, Enterococcus, and Lactobacillus. The phylum Bacteroidetes comprises Gram-negative bacteria, such as Bacteroides, Alistipes, and Parabacteroides. Zhu et al. [35] found that under HS conditions species in the Gram-negative phylum increased while at the same time Firmicutes decreased. This was in contrast to another study finding that Bacteroidetes decreased their abundance in chickens under HS while Firmicutes increased [36]. It can be presumed that microbiome composition is affected by many factors and so it is difficult to predict the effect of temperature on the microbiome. In study with broiler chickens, a phenomenon of decreased butyrate-producing bacteria, such as Faecalibacterium, has been observed [37]. Butyrate is well known for its anti-inflammatory properties [38,39]. It can be proposed that the reducing number of butyrate-producing bacteria plays a crucial role in systemic inflammation caused by HS. Butyrate serves as a source of energy for colonocytes [40] and improves intestinal barrier function due to the stimulation of mucin-producing goblet cells [41]. Barrier function is also improved by stimulation of antimicrobial peptides and β-defensins production by intestinal epithelial cells via activation of mTOR and STAT3 [42]. Furthermore, Isobe et al. [43] report that butyrate promoted T cell-independent IgA production in the colon. HS significantly disrupts intestinal barrier function, and decreased butyrate production in the gut seems to be one of the causative agents for systemic inflammation. A proven effect of HS is increased intestinal permeability and then a greater possibility for pathogenic bacteria to permeate the intestine barrier. In such conditions, increased levels of Salmonella have been observed in the blood and liver of heat-stressed chickens in comparison with a control group [44]. A leaky gut caused by HS is associated with the disrupted function of tight junction proteins. Tabler et al. [45] report that in heat-stressed birds PALS1-associated tight junction protein and cadherin1 were downregulated in the jejunum while occludin (OCLN) and zonula occludens-1 (ZO-1) were downregulated in the ileum. Moreover, HS has been shown to retard intestinal morphology as evidenced by shorter villi [46]. Oxidative stress plays a role in inducing all these negative impacts at the intestine level and additionally can aggravate the effects of toxins and pathogens acting in the intestinal tract [10].

## 4. Oxidative Stress Caused by Heat Stress

Associations between HS and oxidative stress in chickens have been studied since the beginning of this century, when increasing levels of malondialdehyde and activity of catalase, superoxide dismutase, and glutathione peroxidase were determined in chickens exposed to high ambient temperatures [47]. Mitochondria are the source of ROS. Under optimal conditions, about 1–4% of oxygen is oxidized there to superoxide. This can be further reduced to peroxide and an extremely reactive hydroxyl radical in the presence of ferrous and cuprous ions. Therefore, ROS production at a certain level is normal in physiological conditions, and it is neutralized by the body’s antioxidative system. HS, however, can cause ROS overproduction beyond what the physiological antioxidant system of the body can process, and such a state is defined as oxidative stress [48]. After heat exposure, birds increase their respiratory rate, and the body’s supply of oxygen increases. Yang et al. [49] proved that the cells of broilers boost their energy expenditure after heat treatment. Huang et al. [50] reported that electron leakage occurs in electron mitochondrial complexes I and III because their activity is suppressed in heat-stressed chickens. Additionally, uncoupling proteins downregulation after acute HS has been associated with superoxide overproduction in the muscle tissue of young cockerels. The negative effects of HS on avian uncoupling protein expression in the liver tissue of quails also has been described [51]. Uncoupling proteins comprise a group of mitochondrial inner membrane proteins that uncouple respiration from ATP synthesis through the dissipation of the proton gradient [52]. It is well known that cells tolerate mild oxidative stress due to a cascade of antioxidants. While providing new insights, the entire avian antioxidant system was detailed in a review by Surai et al. [53]. Briefly, cell response to oxidative stress can be divided into several levels. First, the antioxidative system maintains the production of ROS by reducing the activities of enzymes responsible for ROS production, such as the NADPH oxidase family. Mitochondria integrity is one of the major points in maintaining manageable levels of oxidant molecules, and disruption of mitochondria integrity is a well-known consequence of HS [50]. Another part of the oxidative system comprises antioxidant enzymes and molecules with radical-scavenging potential. Superoxide dismutase, glutathione reductase, and glutathione peroxidase were upregulated in heat-stressed chickens 21 days old. In chickens 42 days old, on the other hand, all enzymes were downregulated, probably due to exhaustion of the antioxidant system [54]. The activity of antioxidant enzymes seems to be highly dependent upon the period of HS exposure, age, and tissue type [55]. The antioxidant system includes molecules that serve for recycling oxidized molecules of antioxidants. It has been proven that simultaneous supplementation of tocopherol and ascorbic acid is more effective in heat-stressed chickens compared to supplementing tocopherol alone [56]. Oxidative stress can be caused also by cold stress, and HSP plays an important role in protecting protein during periods of low temperatures. Chickens exposed to acute cold stress have been shown to have increased activity of superoxide dismutase in the spleen, thymus, and bursa of Fabricius. On the other hand, glutathione peroxidase had greater activity during chronic cold stress in the spleen and thymus. Members of the HSP family were upregulated in both chronic and acute cold stress groups, thus suggesting their role in the protection of cells during cold periods [57].

## 5. Effect of HS on Chicken Immune Response

As mentioned above, HS creates a pro-inflammatory environment in the body. The key point for starting these processes is the loss of intestinal barrier integrity. Studies have described mechanisms and how HS is involved in these mechanisms. Orally delivered fluorescent isothiocyanate dextran has been observed to increase significantly in the blood plasma of modern fast-growing broiler chickens. At the same time, HS downregulated cadherin1 expression in the jejunum and ZO-1 tight junction proteins in the ileum of broiler chickens [45,58]. Subsequently, increased lymphoplasmacytic inflammatory infiltrates can be observed along the whole intestine [11]. Seventy-two hours after heat exposure, decreased villus height and crypt depth, as well as pathological changes, have been observed in the gut of broiler chickens [46]. A disrupted intestinal barrier is an entry point for pathogens located inside the lumen of the intestine [59]. Pathogens such as Salmonella enteritidis can pass through the intestine wall, migrate to the spleen, and then cause exaggerated inflammation [11]. In another study, Escherichia coli O157:H7 induced an inflammatory response in heat-stressed chickens through TLR 4 receptor, then enhanced expression of NF-κβ, and finally stimulated inflammation by production of pro-inflammatory cytokines IL-1β and TNF-α [60]. Upregulation of IL-6 and IL-8 mRNA expression as inflammatory markers have been observed in the jejunum and ileum of heat-stressed chickens [61]. The inflammatory response then spreads to other parts of the lymphoid tissue. He et al. [62] report that HS induced a strong inflammatory response in chicken spleen through significant upregulation of IL-1β, IL-4, and IL-6 cytokines, as well as NF-κβ molecule expression. Degradation of lymphoid organs, such as the bursa of Fabricius, thymus, and spleen, is typical evidence of HS. The lymphoid organs decrease in weight and pathological findings such as decellularization, blood spots, and disrupted structure of follicles are observed [7]. The inflammatory response is triggered by three classes of transcription factors. The most important transcription factors, NF-κβ and interferon regulatory factors (IRFs), belong to the first of these [63]. Activation of these transcription factors results in the production of pro-inflammatory cytokines, and the most significant of these are IL-1β, IL-6, IL-8, and TNF-α. IL-1β is responsible for the acute phase of inflammation and activation of immune cells in the first wave of inflammation, typically heterophils [64]. IL 6 stimulates the production of acute-phase proteins, such as serum amyloid A and C-reactive protein. Therefore, IL-6 and IL-1β together create an initial phase of inflammation and further activate B and T lymphocytes and hematopoiesis [65]. IL-8 functions as a chemokine in birds, so its function is to attract immune cells to the affected area. Expression of IL-6 and IL-8 in the spleen and cecal tonsils in Eimeria-infected chickens was found to be highly correlated. These two cytokines cooperate in the inflammatory response [66]. TNF-α stimulates a series of inflammatory molecules in cell death signaling pathways and it is divided into transmembrane and soluble forms. The transmembrane form is a precursor converted by TNF-α-converting enzyme to an active, soluble form of TNF-α [67]. The pro-inflammatory immune response seems to be dependent upon the length of the heat exposure period. Chronic HS has been shown to suppress the production of pro-inflammatory cytokines, induce anti-inflammatory cytokines, and support adaptive immunity [68]. A recent study demonstrated the involvement of HSP 60 and HSP 70 in regulating pro- and anti-inflammatory cytokines in chronic HS [28]. The heterophil/lymphocyte (H/L) ratio has been used in many studies as a typical indicator of inflammation. Aengwanich [69] observed an increased H/L ratio in chickens after 7 days of HS. After 14 and 21 days of exposure, however, the H/L ratio decreased, suggesting downregulation of inflammatory response after exposure to HS. Similarly, the antioxidative system can also adapt to HS. Shortly after heat exposure, glutathione peroxidase activity has been observed to increase and then decrease, and after 21 days of heat exposure, it reached the same levels as seen in a thermoneutral control. Malondialdehyde, a common indicator of oxidation in tissues, was found to be increased in response to HS but, subsequently, after 21 days of HS exposure, it diminished to levels as in the thermoneutral control [70]. It should be noted, however, that a decrease in antioxidant enzyme activity could be caused also by the exhaustion of the organism. Therefore, it can be concluded that the initial phase of immune response in HS is accompanied by a strong inflammatory response, but, with a prolonged period of HS, increased production of anti-inflammatory cytokines then occurs. By these mechanisms, the immune system tries to prevent the organism’s exhaustion from a prolonged period of inflammatory response. Prolonged HS can lead to exhaustion of the immune system. Programmed death immunoreceptor (PD1) plays a crucial role in this process. PD1 is expressed in T lymphocytes, B lymphocytes, natural killer cells, and cells of the myeloid lineage [71]. Exhaustion of immune cells has been described in T lymphocytes in cases of chronic viral diseases and cancer, and it presents as suppression of immune response by induction of apoptosis and cell unresponsiveness [72]. These mechanisms have not been described in the heat-stressed organism, but, because stress is related to systemic exhaustion of the organism, immune exhaustion can be considered to play a role during HS.

## 6. Heat Stress and Food Safety in Chickens

The impaired function of the intestinal barrier due to HS represents a potential entrance gate for pathogens. In the USA, the pathogens most responsible for adversely affecting poultry products are *Salmonella*, *Clostridium perfringens* [73], and *Campylobacter* [74]. The season has a significant effect on the prevalence of *Salmonella* in the USA, where a higher probability of contamination was found in the summer months [75]. *Campylobacter* contaminations found in chicken meat were also more abundant in summer [76,77]. Alhenaky et al. [44] found a strong inflammatory response in chickens exposed to HS and infected with *Salmonella*, with resultant invasion into lymphoid tissue and meat. HS also induced changes in intestinal microbiota composition and create a pro-pathogenic environment. *Clostridium* numbers in the intestine of stressed birds increased and the risk of invasion rapidly enhanced with disrupted intestinal morphology [78]. Microbial endocrinology represents another interesting field to study how hormones in direct or indirect ways can influence microbial growth. Corticosterone administration was associated with the enhancement of clostridia representation in the intestine [79]. In another study, corticosterone was shown to alter the production of mucus and tight junction protein expression in chicken intestines. Stress hormone function could be one of the key mechanisms of heat stress-induced susceptibility to pathogens [80]. All these results show season dependence in the prevalence of poultry pathogens outbreaks. Summer weather ensures optimal growth of pathogenic bacteria and HS through deteriorated intestine barrier function further supporting their colonization of poultry products.

## 7. Impacts of Heat Stress on Cells of the Chicken Immune System

### 7.1. Dendritic Cells

Dendritic cells are the main presenting cells of the immune system, and they create a connection between innate and adaptive immunity [81]. In an in vitro experiment by Van Goor et al. [82], chicken bone marrow-derived dendritic cells (BMDDC) were stimulated with heat treatment and with lipopolysaccharide (LPS) to evaluate the response of BMDDC to stimulation by those two stressors. LPS induced higher expression of pro-inflammatory cytokines than did heat. Nitric oxide production was also much more pronounced in the LPS-treated group. On the other hand, HSP was strongly induced in BMDDC during heat treatments. The combination of heat treatment with LPS led to the downregulation of LPS-induced inflammation. Additionally, heat treatment downregulated LPS-induced expression of maturation-related genes, such as MHC-II and CD40, in BMDDC. Taken together, results suggest a role of HSP in downregulating inflammatory response stimulated by BMDDS [82]. Furthermore, another important finding is that the heat did not by itself stimulate an inflammatory response in the in vitro culture. Therefore, HS-induced inflammation is a consequence of disrupted gut barrier function due to a flow of inflammation-inducing agents into the organism and those agents’ stimulating inflammation.

### 7.2. Macrophages

Macrophages are phagocytic cells with antigen-presenting capability. Generally, they are divided into two main subtypes: M1 can rapidly phagocytose and process pathogens; the M2 subtype is responsible for tissue regeneration, such as by transforming into osteoclasts [83]. Chicken macrophages exposed to 45 °C diminished their expression of CCL4, CCL5, IL-1β, IL-8, and inducible nitric oxide synthase below levels seen in the thermoneutral control. On the other hand, HSP expression was highly and significantly expressed in the heat-stressed group. Simultaneous stimulation of macrophages by LPS and heat, by contrast, led to upregulation of pro-inflammatory cytokines IL-1β, IL-8, and inducible nitric oxide synthase [84]. Quinteiro-Filho et al. [85] report that macrophages isolated from the peritoneal cavity of heat-stressed chickens had significantly decreased basal and Staphylococcus aureus-induced oxidative burst. In another study, heat-stressed macrophages in vitro had pronounced lower production of inflammatory mediators due to higher production of anti-inflammatory cytokine IL-10 [86]. Zhang et al. [87] have reported that negative impacts of HS on macrophage function can be seen in osteoclasts. Systemic inflammation caused by HS can stimulate higher numbers and activity of osteoclasts, which in turn cause higher resorption of bone mass and potentially induce skeletal damage in chickens.

### 7.3. B Lymphocytes

Two major sites of B lymphocytes in birds are the bursa of Fabricius and the spleen. In the bursa of Fabricius, they comprise at least 95% of the entire cell’s population. In the spleen, they are in germinal centers together with follicular dendritic cells and their population makes up less than 20% of the whole organ. During HS, however, both the lymphoid organs tend to decrease in weight. The percentage of B lymphocytes is not influenced by HS in either organ [7]. In peripheral blood, B lymphocytes have been shown to decrease in heat-stressed birds. Additionally, heat treatment early in chickens’ lives decreased the numbers of B lymphocytes in the blood after vaccination against the Newcastle disease virus. However, IgM and IgG levels in the blood increased with heat treatment to levels above those in vaccinated birds. Heat treatment early in chickens’ lives can influence the humoral immune response in the later stages of broiler fattening [88]. Consistently with the just-cited study, Tang and Chen [89] observed significantly decreased numbers of B lymphocytes in peripheral blood and the bursa of Fabricius. IgM, IgG, and IgA levels in the blood were significantly decreased in heat-stressed chickens [89]. Wu et al. [90] also reported their findings that the numbers of proliferating B cells were significantly decreased in peripheral blood during a period of HS. Reduced numbers of B lymphocytes can be explained by structural damage and heat-induced apoptosis in the bursa of Fabricius [91]. Studies of this nature predominantly have described decreasing numbers of B lymphocytes. Consistent with this, many studies have determined lower levels of antibodies because of HS. Mashaly et al. [6] reported that antibody titers in commercial laying hens decreased significantly in a chronically heat-stressed group but not in hens under cyclic HS. Hirakawa et al. [7] found that plasma titers of IgY, IgM, and IgA antibodies against bovine serum albumin were lower in heat-stressed broilers than in thermoneutral control animals. Bartlet and Smith [92] report that primary and secondary antibody response to sheep red blood cells was also negatively affected by HS, and Jahanian and Rasouli [93] published that antibody response against infectious bronchitis virus was diminished by HS in broiler chickens. Experiments have shown that HS causes structural damage to the bursa of Fabricius, a major site of B lymphocytes in chickens, and subsequently decreases their numbers in the blood. Consequently, antibody response to various antigens is disrupted in heat-stressed chickens.

### 7.4. T Lymphocytes

The thymus is a major site for the development of T lymphocytes; these cells are typically divided into two main subtypes: CD8^+^ cytotoxic and CD4^+^ helper T lymphocytes. Mature T lymphocytes are CD3^+^, but CD3^+^ T lymphocytes can be found as CD4^+^CD8^+^ and CD4^−^CD8^+^ and αβ CD4^−^CD8^−^ and γδ CD4^+^CD8^−^ subtypes [94]. It is noteworthy that HS has been shown to cause an increased percentage of immature CD4^−^CD8^−^ and suppression of CD4^+^CD8^+^ lymphocytes in the chicken thymus [7]. Several studies have evaluated the effects of HS on CD4 helper and CD8 cytotoxic T lymphocytes. Total T lymphocyte numbers (CD3^+^) and subtypes CD4 and CD8 increased after a period of HS in the first 6 days of broiler chickens’ lives [88]. When chickens were exposed to HS every day for 2 h, CD3, cytotoxic, and helper T lymphocyte numbers decreased, but CD4 helper T lymphocytes are more affected. Similarly, Jahanian and Rasouli [93] reported that in chickens’ peripheral blood CD4 cells were significantly decreased and CD8 increased in a period of chronic HS. In another study, while HS decreased CD4 and CD8 in the blood of chickens, in the spleen CD4 decreased and CD8 significantly increased. In that same experiment, by Trout and Mashaly [95], other chickens received adrenocorticotropic hormone with almost the same levels of CD4 and CD8. Changes in the composition of T lymphocyte subsets can be explained in part by the effects of stress hormones, and it has been reported that exposure to corticosterone inhibits the proliferation of lymphocytes [96]. However, more studies must be performed to confirm the ways in which HS influences the presence of individual T lymphocyte subtypes.

### 7.5. Heterophil/Lymphocyte Ratio

Heterophils play a crucial role in innate immunity. Inasmuch as they carry out phagocytosis, these cells represent a mechanism in the first line of immune defense after infection. Heterophils level is a well-known indicator of inflammation, and the H/L ratio is widely used as an indicator of HS. The initial phase of the inflammatory response to HS is accompanied by heterophil activation and their increasing numbers are found in blood. Soleimani et al. [97] proved this response by observing increasing H/L ratio in three different chicken breeds with various levels of susceptibility to HS when exposed to acute HS. In another study, Lee et al. [98] observed an increasing H/L ratio in laying hens exposed through 8 weeks to multiple stress conditions. The H/L ratio has been tested as an indicator for several purposes. In domestic fowl, H/L was identified as an indicator of susceptibility to HS [99]. Additionally, the ratio can be used as a biomarker of susceptibility to Salmonella enteritidis infection in chickens [100]. In a recent study, Wang et al. [101] used a genome-wide profiling technique to identify genes and pathways associated with the regulation of the H/L ratio in chickens. They identified the gene C1QBP as an important candidate gene in regulating the H/L ratio. This gene is involved in protecting cells against oxidative stress-induced apoptosis.

## 8. Baicalin and Baicalein

Baicalin and baicalein are flavonoids isolated from the root of Scutellaria baicalensis and have proven anti-inflammatory [102,103] and antioxidant [104] properties. Baicalin is a glycosylated form of baicalein. Immunomodulatory properties of both baicalin and baicalein have been confirmed by many models. Predominantly anti-inflammatory effects of baicalin and baicalein have been described. Baicalin was shown to reduce the expression of TLR 2, TLR 4, inducible nitric oxide synthase, cyclooxygenase-2, and NF-κβ in rat brain and additionally to attenuate the serum content of TNF-α and IL-1β [105]. In vitro epithelial cell culture, Dong et al. [106] observed baicalin to decrease LPS-induced IL-6, IL-8, and TNF-α mRNA expression and levels measured by ELISA. Additionally, baicalin suppressed the expression of NF-κβ. Kim et al. [107] have reported that in RAW 264.7 mouse macrophages, baicalein was able to inhibit nitric oxide, IL-1α, and IL-6 production after polyinosinic–polycytidylic acid-induced inflammation. In another study, baicalein mitigated inflammation in periodontal ligament cells by attenuation of IL-1β, TNF-α, and monocyte chemoattractant protein 1 production and through mitogen-activated protein kinase (MAPK) signaling inhibition [108]. Both baicalin and baicalein have been found to mitigate LPS-induced vascular injury. They inhibited LPS-induced vascular barrier disruption, expression of cell adhesion molecules, and migration of monocytes to endothelial cells. All this suggests that the molecules can inhibit the entire process of inflammation. Taken together, baicalin and baicalein have anti-inflammatory effects that could be helpful in cases of HS-induced systemic inflammation [109]. Both phytochemicals have strong antioxidant abilities. Baicalin was tested for its antioxidant activity and compared with ascorbic acid and butylated hydroxytoluene, which are commonly used antioxidants. Baicalin was found to have radical-scavenging activity greater than that of butylated hydroxytoluene but lower than that of ascorbic acid. Electron-donating ability was tested by the Fe (III) reduction method, which showed baicalin to have stronger reducing power in comparison to both ascorbic acid and butylated hydroxytoluene. Iron-chelating ability is important in preventing the reduction of hydrogen peroxide to hydroxide radicals by binding to Fe^2+^. Iron chelating activity in baicalin was slightly lower than that of ascorbic acid and hydroxytoluene [110]. In another study, the antioxidant activity of four main flavones (baicalin, baicalein, wogonoside, and wogonin) from *S. baicalensis* were tested and compared. Baicalein was three times stronger than baicalin in free radical-scavenging activity. The reducing power of baicalein was also significantly greater than that of baicalin, and, in a linoleic acid peroxidation assay, baicalein was pronounced to have the best antioxidant ability among all tested flavones [111]. Similarly, baicalein bound to ferrous ions more strongly than did ferrozine, a strong iron chelator. Therefore, baicalein inhibited Fenton’s reaction by a combination of iron-chelating activity and radical scavenging properties. Baicalin provided only partial antioxidant effects in comparison with baicalein [112]. As described in a review by de Oliveira et al. [113], mitochondria, as a source of ROS, are influenced by baicalin and baicalein. Briefly, baicalin magnifies ATP production and citrate synthase activity. Additionally, it decreases ROS production and lipid peroxidation in membranes of mitochondria, activates PGC-1α, and stimulates higher expression of Mn-superoxide dismutase and glutathione peroxidase [113]. Baicalein is involved in protecting against apoptosis. Its protective effect against oxidative stress-induced apoptosis has been demonstrated because baicalein treatment protected cells against 6-hydroxydopamine and hydrogen peroxide-induced production of ROS and apoptosis [114,115].

### 8.1. Baicalin and Baicalein and Their Proven Effects on Avian Models

Studies of avian models have proven strong anti-inflammatory effects of baicalin in experiments against various stressors. The anti-inflammatory properties of baicalin using avian models are demonstrated in Table 1. Although the impacts of baicalein on the stressed poultry organism have not been studied as much as in the case of baicalin, some studies have shown similar effects with baicalin and proven its anti-inflammatory and antioxidative potential. Studies with baicalein using avian models are shown and summarized in Table 2.

### 8.2. Using Baicalin and Baicalein to Mitigate Heat Stress in Chickens

As described above, baicalin and baicalein have consistently proven to have anti-inflammatory and anti-oxidative effects in poultry. Their use can be beneficial under the conditions of HS. The two compounds can be supplemented synergistically in HS conditions. Varmuzova et al. [37] supplemented chickens with 0.2% of Scutellaria baicalensis (SB) extract and exposed chicken for 2 days to 35 °C or infected by Salmonella enteritidis. mRNA expression of inflammatory markers was the same in heat-treated chickens supplemented with S. baicalensis as in control birds. A mixture consisting of extracts of SB and Curcuma significantly decreased Salmonella counts in the caecum and protected chickens against salmonella-induced inflammation. A possible mode of action could relate to changes in microbiota composition within the caeca. In those chickens supplemented with SB extract, there was greatly increased mRNA expression of Faecalibacterium, a butyrate-producing commensal bacteria with a proven anti-inflammatory effect [116]. Active compounds of SB can influence immune response, but an appropriate concentration must be applied. Króliczewska et al. [117] observed some negative effects on the immune system and lymphoid tissue in chickens supplemented with dried roots of SB. With increasing concentrations in the diet (0.5%, 1.0%, and 1.5%), dried SB roots increased the H/L ratio in blood and decreased the weight of the spleen and bursa of Fabricius. Therefore, extracts seem to offer a better way of supplementation than dried roots. Baicalin and baicalein have many beneficial effects for heat-stressed chickens, and their use as a supplement in periods of the year when HS occurs could be a milestone in mitigating a major stressor in today’s poultry production.

**Table 1:** Anti-inflammatory effects of baicalin against inflammation-inducing agents in avian models.

Administration	Stressor	Model	Effect of Baicalin Administration
Oral gavage	Avian pathogenic *Escherichia coli*	Hy-line brown chickens	Decreased death rate. Inhibition of NF-κβ signaling. Decreased myeloperoxidase activity. Downregulation of IL-1β, TNF-α, IL-6 cytokines [118].
Oral gavage	Lipopolysaccharide-induced liver inflammation	Beijing white chickens	ELISA and qPCR confirmed dose-dependent decrease in IL-1β, IL-6, and TNF-α production. Suppressed mRNA expression of inducible nitric oxide synthase, cyclooxygenase 2, TLR 4. NF-kβ pathway inhibition [119].
Powder in feed	H9N2 Avian influenza virus	SPF chickens	Replacement of secondary *Escherichia coli* infection in gut by increase in *Lactobacillus* strain. Inhibition of intestinal damage and tight junctions. Lower serum malondialdehyde. Suppression of mRNA expression of IFN-γ, TNF-α, IL-22, IL-17A, IL-6, and IL-1β [120].
Allantoic cavity injection of chicken embryos	*Mycoplasma gallisepticum*-induced lung inflammation	Chicken embryos	Alleviation of lung pathological changes. ELISA and RT-qPCR proved downregulation of IL-1β, IL-6, and TNF-α cytokines. Inhibited expression of TLR 6, MyD88, and NF-κβ and nuclear translocation of NF-κβ-p65 [121].
Oral gavage	*Mycoplasma gallisepticum*	White leghorn chickens	Reduced *Mycoplasma gallisepticum* colonization. Reversed peripheral accumulation of phenylalanine induced by *M. gallisepticum*. Enriched the commensal bacterium *Bacteroides fragilis* in the gut. Decreased expression of TNF-α, IL-6, IL-8, and IL-1β [122].
Oral gavage	*Mycoplasma gallisepticum*	White leghorn chickens	Increased ATPase activities and mRNA and protein expression level of energy metabolism-related genes. Attenuated apoptosis in chicken lung. Decreased TNF-α and IL-1β cytokines [123].
Oral gavage	*Mycoplasma gallisepticum*	White leghorn chickens	Alleviated apoptosis in bursa of Fabricius. Decreased expression of TNF-α and NF-κβ. Increased numbers of CD8^+^ cells in bursa of Fabricius. Decreased expression of autophagy-related genes [124].
Oral gavage	*Mycoplasma gallisepticum*	Leghorn chickens	Alveolar type I epithelial cells injury was reduced. Decreased levels of IL-1β, TNF-α, and TLR 2 expression [125].
Oral gavage	*Mycoplasma gallisepticum*	White leghorn chickens	Upregulation of nuclear factor erythroid 2-related factor 2 signaling pathway to counteract oxidative stress in thymus. Decreased apoptosis-related genes and proteins in thymus. Decreased TNF-α, IL-6, IL-8, and IL-1β cytokines [126].
Oral gavage	*Mycoplasma gallisepticum*	White leghorn chickens	Upregulation of nuclear factor erythroid 2–related factor 2 and heme oxygenase-1 pathway. Suppression NF-κβ pathway in the spleen. Increased catalase and superoxide dismutase activity. Decreased levels of malondialdehyde, nitric oxide, and inducible nitric oxide synthase. Downregulated TNF-α and NF-κβ, as well as caspase 3, caspase 9, and Bax protein [127].
Mixed in feed	Avian pathogenic *Escherichia coli*	Hy-line brown laying hens	Elevating zona occludens, claudins, occludin, avian β-defensins, lysozyme mRNA levels and ZO-1, claudin1, and occludin protein levels. Elevated superoxide dismutase catalase, and glutathione peroxidase mRNA expression. Suppressed TNF-α, IL-1β, IL-6, and IL-8 cytokines. Upregulation of mRNA levels of anti-inflammatory cytokines IL-4, IL-10, IL-13, and TGF-β [128].
Intragastric administration	Zearalenone	Jute chicks	Decreased aspartate aminotransferase, alanine aminotransferase, and creatinine levels in plasma. Ameliorated pathological observation in kidney and liver. Inhibition of TNF-α, IL-1β, and cyclooxygenase-2, with caspase-3 and caspase-11 [129].

**Table 2:** Effects of baicalein described in poultry.

Administration	Stressor	Model	Effect of Baicalein Administration
Feed supplement (optimal dosage: 100 to 200 mg/kg)	-	Arbor Acres broiler chickens	Increased body weight and body weight gain. Increased CD3^+^/CD4^+^ and CD3^±^/CD8^+^ ratios. Decreased ratios of non-HDL-C/HDL-C, LDL-C/HDL-C, TC/HDL-C, triglycerides, and LDL. Increased activity of SOD, GSH-Px, and CAT in plasma. Increased T-AOC activity, T-SOD, and GSH-Px levels in liver [130].
Added to in vitro culture	Hypoxia	Chicken cardiomyocytes	Reduced hypoxia–reoxygenation-induced myocardial death and apoptosis [131].
Inoculation of chicken embryos through the allantois cavity	Bursal disease virus	Embryos	Dose-dependent inhibition of NF-κβ pathway activation and TNF-α and IL-1β cytokines expression. Dose-dependent decrease in histamine concentration in liver [132].
Feed supplement	-	Hubbard × Cobb-500 broiler chicks	Reduced breast muscle and subcutaneous and abdominal fat weights. Higher expression of mRNAs for genes encoding factors involved in adipogenesis and fat storage, 1-acylglycerol-3-phosphate-O-acyltransferase 2, CCAAT/enhancer-binding protein β, perilipin-1, and sterol regulatory element-binding transcription factor 1 [133].

## 9. Conclusions

HS causes overproduction of ROS and the resulting oxidative stress has deleterious effects on tissues and stimulates inflammation. Depending upon the period of exposure, HS-induced inflammation and ROS have deleterious effects on the productive performance of chickens, on their immune systems, and on the organism’s overall ability to defend itself. Reduction of oxidative stress and inflammation seems to be crucial for the alleviation of heat stress. Baicalin by its anti-inflammatory and immunosuppressive effects can decrease the production of pro-inflammatory cytokines and decrease the recruitment of cells involved in inflammation. Baicalein as a strong antioxidant by radical scavenging and chelating effects can protect the cells against overproduction of ROS during HS. Therefore, based on the literature, both flavonoids represent mitigation strategies to alleviate the adverse effects of HS in poultry. It is important to mention that the negative impacts of heat stress are mitigated by ensuring a proper environment in husbandry, sanitation and feeding technique and feeding additives represent supporting methods to improve the health and performance of broiler chickens. However, baicalin and baicalein supplementation can improve the function of the gastrointestinal tract and immune system to improve hen performance and profitability of broiler production in stressful conditions. For future directions, it is necessary to optimize the method of administration and concentration and ensure the stability of flavonoids in the environment for their use as an additive.

## Data Availability

Not applicable.
